# Design of novel substituted phthalocyanines; synthesis and fluorescence, DFT, photovoltaic properties

**DOI:** 10.3906/kim-2007-40

**Published:** 2020-12-16

**Authors:** Mehmet Salih AĞIRTAŞ, Derya GÜNGÖRDÜ SOLĞUN, Ümit YILDIKO, Abdullah ÖZKARTAL

**Affiliations:** 1 Department of Chemistry, Faculty of Science, Van Yüzüncü Yıl University, Van Turkey; 2 Architecture and Engineering Faculty, Department of Bioengineering, Kafkas University, Kars Turkey; 3 Department of Physics, Faculty of Science, Van Yüzüncü Yıl University, Van Turkey

**Keywords:** Fluorescence, phthalocyanine, synthesis, DFT, photovoltaic

## Abstract

The 4-(2-[3,4-dimethoxyphenoxy] phenoxy) phthalonitrile was synthesized as the starting material of new syntheses. Zinc, copper, and cobalt phthalocyanines were achieved by reaction of starting compound with Zn(CH3COO)2, CuCl2, and CoCl2 metal salts. Basic spectroscopic methods such as nuclear magnetic resonance electronic absorption, mass and infrared spectrometry were used in the structural characterization of the compounds. Absorption, excitation, and emission measurements of the fluorescence zinc phthalocyanine compound were also investigated in THF. Then, structural, energy, and electronic properties for synthesized metallophthalocyanines were determined by quantum chemical calculations, including the DFT method. The bandgap of HOMO and LUMO was determined to be chemically active. Global reactivity (I, A, η, s, μ, χ, ω) and nonlinear properties were studied. In addition, molecular electrostatic potential (MEP) maps were drawn to identify potential reactive regions of metallophthalocyanine (M-Pc) compounds. Photovoltaic performances of phthalocyanine compounds for dye sensitive solar cells were investigated. The solar conversion efficiency of DSSC based on copper, zinc, and cobalt phthalocyanine compounds was 1.69%, 1.35%, and 1.54%, respectively. The compounds have good solubility and show nonlinear optical properties. Zinc phthalocyanine gave fluorescence emission.

## 1. Introduction

There are many reasons that make phthalocyanine compounds interesting. One of them that stands out is the 18 π electron richness related to the chemical structure [1]. This electronic structure adds interesting properties to the compound by providing delocalization [2]. In this context, solar cells [3,4], nonlinear optics [5], chemical sensors [6], liquid crystals [7], laser dyes [8], photocatalytic [9], catalytic [10], photovoltaic [11], photochromic [12], and photodynamic therapy (PDT) [13,14] are being investigated extensively for obtaining photosensitive agents. Despite these wide uses, there are problems with phthalocyanines that need to be investigated. One of these problems is that these compounds do not dissolve to the desired level in water and most organic solvents [15]. To solve this problem, the most effective method of improving resolution is the chemical bonding of substituted groups suitable for peripheral or axial positions. When the solubility problem is overcome, these compounds can potentially be used in many fields (such as photosensor agents in PDT) [16]. The focus of research to solve this problem is the use of new original substituent groups. Recently, researchers have been focusing on the use of phthalocyanine compounds in dye-sensitive solar cells. Studies in this field are seen as an alternative for clean energy and a clean environment. Compounds that provide high power conversion efficiency are regarded as an effective candidate for dye-sensitive solar cells [16]. Therefore, the synthesis of new types of phthalocyanine complexes that are sensitive to dye is necessary [16,17]. It helps to obtain different alternatives for clean and renewable energy with the synthesis of suitable compounds. Also, phthalocyanines have lower economic costs than sensitizers such as ruthenium used for a dye-sensitized solar cell.

Herein, fluorescence, aggregation, photovoltaic performance properties, and synthesis of tetra 3,4-dimethoxyphenethoxyphenoxy phthalocyaninato complexes were reported. In addition to the experimental studies of synthesized metal complex molecules, geometry, conformational stability, and electronic properties have been investigated. For this purpose, in this study, we defined the molecular structure, nonlinear optic (NLO) analysis, molecular electrostatic potential (MEP) maps, and molecular surface properties. Quantum chemical studies were calculated according to a 6-311 G basis set in the ground state and DFT/B3LYP levels. Furthermore, HOMO and LUMO energies, total energy, ΔE (LUMO-HOMO) energy gap, and global reactivity descriptors (I, A, η, s, μ, χ, ω) were calculated. For the phthalocyanines used here, the high power conversion efficiency was found at a reasonable performance level.

## 2. Experimental section

All information about the used equipment, materials, synthesis, theoretical analysis, and photovoltaic experiments is given in the Supplementary Information.

## 3. Results and discussion

### 3.1. Synthesis and characterization

The synthesis reactions of the 4-(2-[3,4-dimethoxyphenethoxy] phenoxy) phthalonitrile (3) and zinc (II), cobalt (II), and copper (II) phthalocyanines are shown in Scheme 1. Synthesis of 4-(2-[3,4-dimethoxyphenoxy] phenoxy) phthalonitrile (3) was carried out by nitro groups of nitrophenol and 4-nitrophthalonitrile compounds displacement reaction with 2-(3,4-dimethoxyphenyl) ethanol. Literature methods with minor modifications were used in the synthesis of the phthalonitrile derivative [18]. The synthesis of 4-(2-[3,4-dimethoxyphenoxy] phenoxy) phthalonitrile was carried out in a basic medium. Dry K2CO3 was used to make the reaction medium basic. K2CO3 is widely used for this type of reaction [19,20]. The reaction was monitored and followed by thin-layer chromatography. The terminated reaction was precipitated with water and isolated. This product was used as the starting compound in the preparation of zinc, cobalt, and copper phthalocyanines. Phthalocyanine complexes synthesized using this starting material dissolve easily in CH2Cl2, CHCl3, THF, DMF, and DMSO. Phthalocyanine complexes, which are easily soluble in solvents, can be used more in applications. One of the obstacles in applications of phthalocyanine compounds is that they are poorly soluble. To solve this problem, axial, peripheral, and nonperipheral groups are added to the phthalocyanine structure [15,21]. If the groups mentioned for solubility also prevent aggregation, they provide an advantage for applications.

The phthalocyanine complexes (4–6) were obtained by the reaction of the template cyclotetramerization of 4-(2-[3,4-dimethoxyphenethoxy] phenoxy) phthalonitrile (3) with metal salts (Zn[CH3COO]2, CuCl2, and CoCl2). The characterization of these compounds was carried out with the help of mass, FT-IR, 13C-NMR, 1H-NMR, and UV-Vis spectra.

As expected from the 1H-NMR spectrum for compound 3, aromatic protons were observed around 8.12–6.90 ppm. It was determined that aliphatic protons of this compound appeared at 4.34, 3.72, 3.70, 3.31, and 2.95 ppm.

In 13C NMR spectrum of phthalonitrile 3 at 162.27, 160.71, 149.96, and 147.96 C=O, at 136.77–136.17 and 130.40–120.55 ppm C=C and at 116.72–116.67 ppm C≡N, at 70.13 ppm CH2 and at 55.99–55.92 ppm CH3 peaks were observed. These data support the expected structure. The 1H and 13C-NMR spectra of compound 3 are shown in Figures S1 and S2. The 1H-NMR spectrum in the DMSO solvent of compound 4 also supports the construct as expected. Aromatic protons are observed as approximately 9.22, 8.81–6.85 ppm, while aliphatic protons appear at 4.73, 3.76, 3.70, 3.41, 3.36, 3.31, 3.25, 2.48, and 1.34 ppm. This phthalocyanine compound is compatible with the expected structure, except for minor chemical shifts relative to the starting material. Based on the literature information on compounds 5 and 6, 1H-NMR measurements were not made. Generally, the types of phthalocyanine complexes carrying the paramagnetic metal atom are excluded from the 1H-NMR spectra [22,23].

Vibration bands of functional groups were observed in FT-IR spectral measurements as expected; 3080 (Ar–CH), 2949–2835 (CH3), 2231 (C≡N), 1600–1517 (C=C), 1249 cm–1 (Ar–O–Ar) confirms the structure of the synthesized phthalonitrile. After the conversion of 4-(2-[3,4-dimethoxyphenethoxy] phenoxy) phthalonitrile 3 into phthalocyanines 4–6, the sharp peak for the (C≡N) vibrations disappeared. The IR spectra of compound 4 displayed aromatic CH peaks at 3100 cm–1 (Ar–H), 2964–2835 cm–1 (CH3), 1598 cm–1 (C=C), Ar–O–Ar peaks at 1261 cm–1. The IR spectra of compound 5 displayed aromatic CH peaks at 3078 cm–1 (Ar–H), 2927–2831 cm–1 (CH3), 1606–1514 cm–1 (C=C), Ar–O–Ar peaks at 1261 cm–1. Similarly, The IR spectra of compound 6 displayed aromatic CH peaks at 3080 cm–1(Ar–H), 2966–2879 cm–1 (CH3), 1600–1514 cm–1 (C=C), Ar–O–Ar peaks at 1259 cm–1. The IR spectrum values of these phthalocyanine compounds and starting material are consistent with similar functional groups in the literature [17,24]. These originally prepared phthalocyanine compounds, besides their high efficiency, are economically important to obtain in a short period of time. Confirmation of spectrally determined compounds with computational chemistry also helps to determine their electronic properties. Phthalocyanine compounds are researched for their rich electronic structures for many technological applications. In accordance with these purposes, it is necessary to investigate factors such as fluorescence, aggregation, and solubility.

### 3.2. Fluorescent spectra

**Figure 1:**
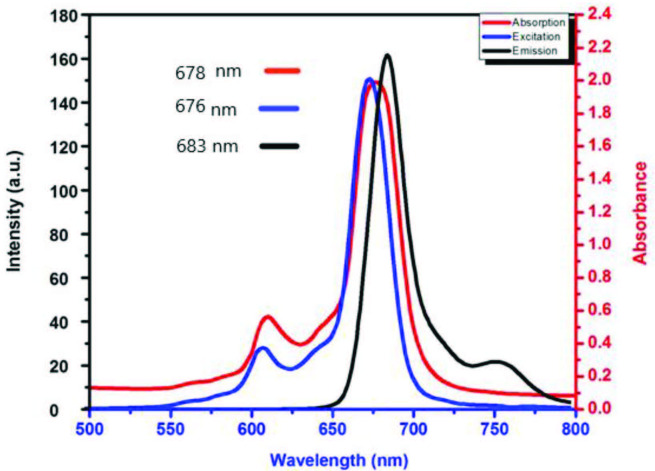
Emission, excitation, and absorption spectra of phthalocyanine compound 4 in THF.

In today’s technology fluorescent compounds have important uses such as disease diagnosis and treatment [25]. Sensor materials such as biomarking [25], environmental indicators [26], enzyme substrates [27], cell-organelle marking [28], radiation-emitting diodes [29], chemistry [30], molecules biology [30], and physics [31] have become an integral part of science. Fluorescence excitation, absorption, and emission spectra of the zinc phthalocyanine compound, which shows fluorescence from these compounds, were examined in THF solvent. Figure 1 shows these spectra. Stokes shifts observed for this compound fall within the appropriate range for phthalocyanines. Stokes shift values of the fluorescence zinc phthalocyanine vary depending on the solvent effect. This value was 7 nm for THF. Fluorescence excitation and emission values of phthalocyanine compound 4 values were found to be in accordance with the values in the literature [32]. The fluorescence activity of this compound allows it to find application potential in the abovementioned areas.

### 3.3. Electronic absorption spectra

UV-Vis spectroscopy is one of the basic devices for phthalocyanine chemistry. Phthalocyanines have specific electronic transitions thanks to 18 π electrons. These transitions are characterized by the Q band in the visible region of the spectrum, which results from π–π * electron transitions from the highest occupied molecular orbitals (HOMO) (a1u and a2u) to the lowest unoccupied molecular orbitals (LUMO), and occurs at about 600–700 nm. The other one is called the B band and can be seen in the 300–400 nm range due to deeper π HOMO to LUMO energy levels [33]. In this study, characteristic data for phthalocyanine compounds were obtained as expected in electronic structure. The zinc phthalocyanine complex gives a Q band in THF solvent at 676 nm and shoulder at 610 nm; similarly, the cobalt phthalocyanine complex gives a Q band at 664 nm at THF. The copper phthalocyanine complex gives a Q band in the same solvent at 676 nm and a shoulder at 610 nm. Zinc and copper phthalocyanines display B bands at 348 and 342 nm, respectively. The fact that phthalocyanines have characteristic electronic transitions in the UV visible region enables phthalocyanines to reveal other properties of UV rays such as DNA binding, DNA photocleavage, and antioxidants. This richness of electronic behavior also makes attractive the investigation of the photovoltaic behavior of these compounds.

### 3.4. Aggregation studies

**Figure 2:**
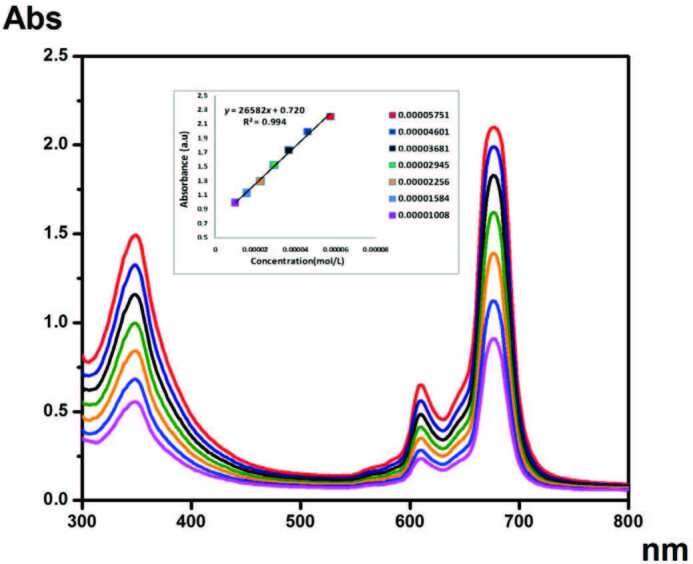
Electronic absorption of compound 4 in different concentrations.

**Figure 3:**
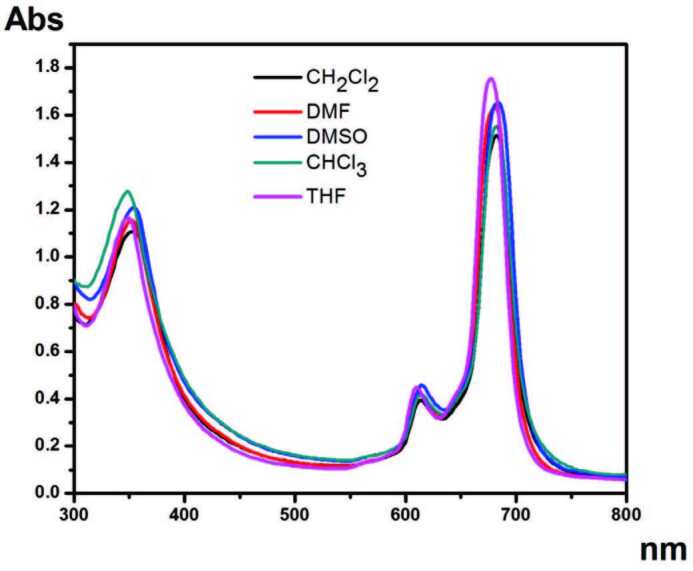
Electronic absorption of compound 4 in different solvents.

Phthalocyanines continue to exist as many different research subjects. Essentially, there are two considerations required to overcome two important hurdles that phthalocyanine compounds face in applications. One of these is to improve the solubility of these compounds. The other is to prevent aggregation in the solvent environment. Nonaggregate phthalocyanine compounds are preferred for many applications. To investigate how these compounds behave in this solvent medium, their aggregation properties are investigated. H and J aggregates are frequently investigated in phthalocyanine chemistry. These studies reveal the behavior of the compound in the solvent [34]. The concentration-related changes of zinc, cobalt, and copper phthalocyanine compounds with electronic absorption in THF solvent are given in Figures 2 and S3–S4. In addition, the electronic absorption of these phthalocyanine compounds in different solvents to determine the effect of different solvents is given in Figures 3 and S5–S6. The concentration results in which the compounds were studied show that the compounds are not aggregated. It is known that nonaggregated phthalocyanine compounds are preferred in promising treatments such as photodynamic therapy. While phthalocyanines generally tend to aggregate in the solvent medium, the nonaggregation behavior of these compounds makes them valuable.

### 3.5. Geometric optimization and structural analysis

**Figure 4:**
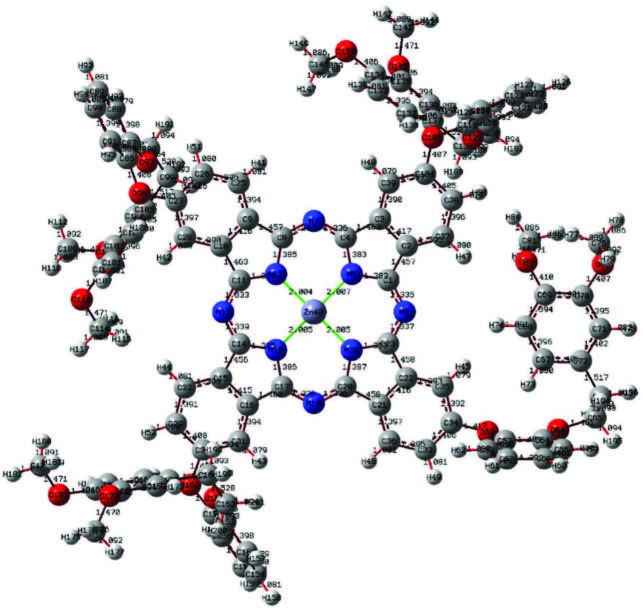
Optimized molecular structure of compound 4 at DFT/B3LYP/6-311G basis set.

**Figure 5:**
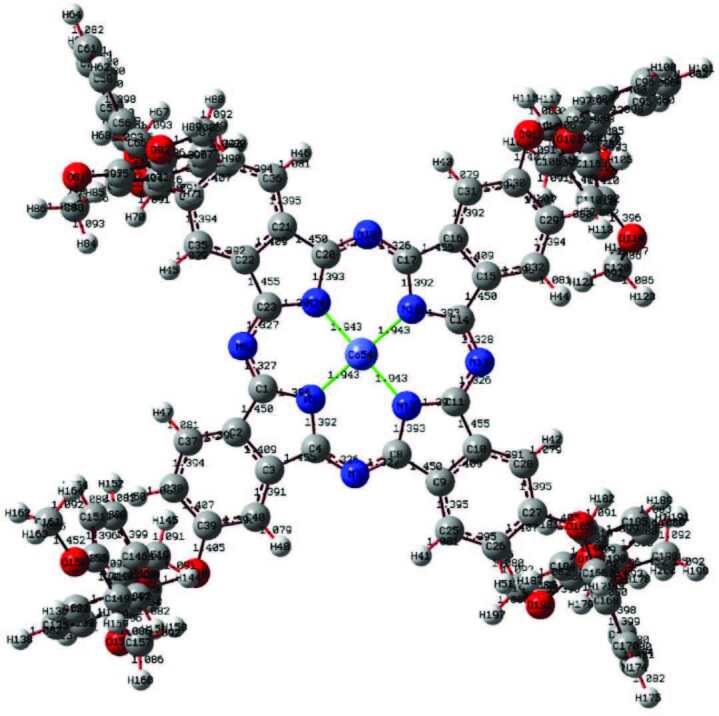
Optimized molecular structure of compound 5 at DFT/B3LYP/6-311G basis set.

**Figure 6:**
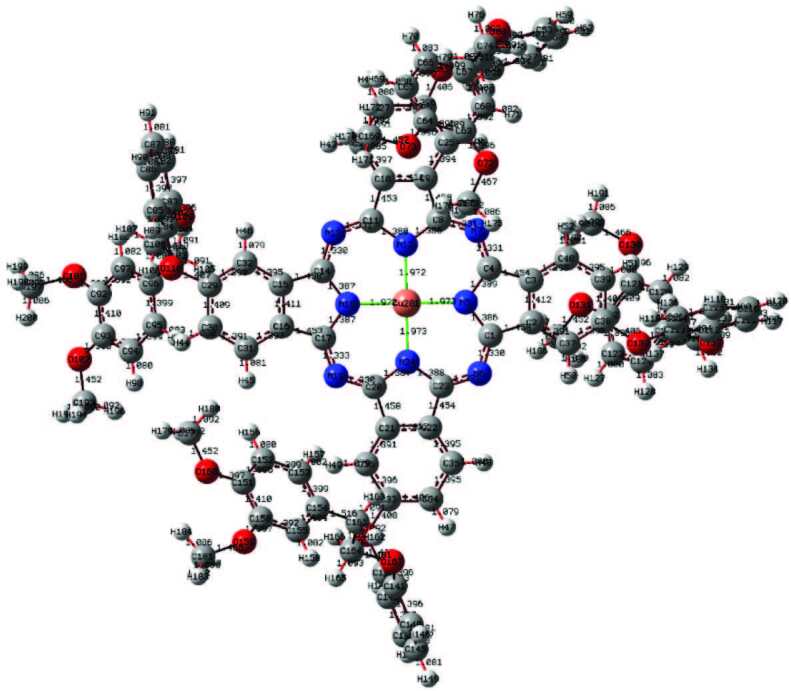
Optimized molecular structure of compound 6 at DFT/B3LYP/6-311G basis set.

In this study, quantum chemical calculation of phthalocyanine compounds having a molecular structure with three different metal centers was performed by a DFT/B3LYP method, which contains a 6-311G basis set. The optimized structures are given in Figures 4–6. Parameters such as planar state, bond angles, and bond lengths of the phthalocyanine core were determined by optimization. In the optimization of the central atom, Zn–N5 2007, Zn–N18 2005, Cu–N5 1.973 Å, Cu–N18 1.972 Å, Co–N5 1.943 Å, and Co–N18 1.943Å were calculated. The bond lengths in the phthalocyanine core vary according to the size of the central atom. However, the values of the C4–N7 bond length are 1.331Å in Zn–Pc, 1.326 Å in Cu–Pc, and 1.336 Å in Co–Pc, and are very close to each other. The fact that the central atom is different does not have much effect on the change of other parameters. With the calculations, it has been determined that Zn41–N18–C14–C15 and C20–N19–C17–C16 atom groups have dihedral angles close to 180°. These angles show that the phthalocyanine core is planar. These parameters contribute to the determination of the molecular structure [35].

**Figure 7:**
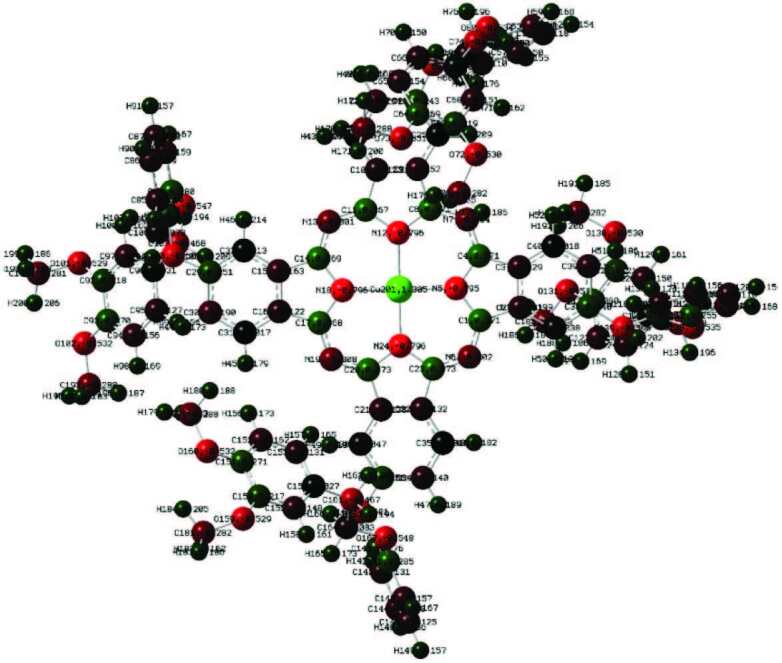
Mulliken atomic charges were calculated with DFT ab-initio B3LYP/6-31G (d, p).

Mulliken atomic charges are orbital-based. Electronic charges in a molecule are collected according to the charge contributions from the orbitals. When determining the charge of an atom, the clouds of electrons overlapping between two atoms are calculated by dividing the two atoms equally [36]. Mulliken charge distribution for zinc atom, respectively; Zn41 (1.493), Cu (1.305), and Co54 (1.295), and nitrogen bound to the central atom were calculated in three phthalocyanines, N12 (–0.796), N24 (–0.795), respectively. Some C atoms in the phthalocyanine nucleus are positive and others are negative. Mulliken atomic charges are shown in Figure 7.

### 3.6. Energetic properties

**Figure 8:**
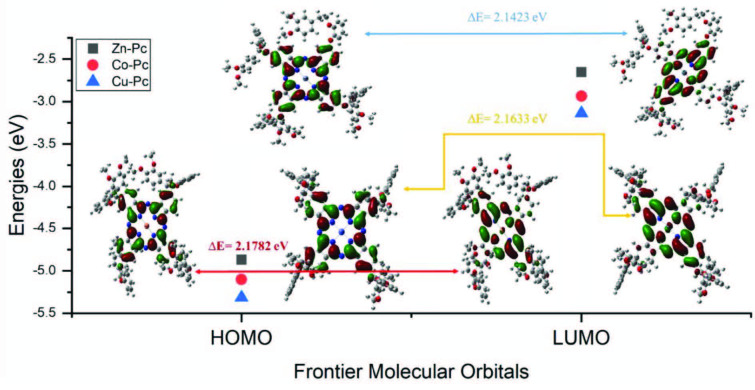
Frontier molecular orbitals of 3,4-dimethoxyphenethoxy) phenoxy substituted metallophthalocyanines by DFT/B3LYP with 6-311 G basis sets.

The energy levels of HOMO-LUMO orbitals were calculated with a 6-311 basis set of DFT-B3LYP method under the Gaussian 09 package program and are shown in Figure 8 [37]. The electronic properties of the optimized compounds 4, 5, and 6 were visualized using the GausView 6.0 program. The results are reported here and the orbital maps of the HOMO and LUMO energies are shown in Figure 8. Molecule for Zn–Pc; EHOMO = –4.8690 eV – ELUMO = –2.6548 eV, calculated. Molecule for Cu–Pc; EHOMO = –5.3147 eV – ELUMO = –3.1364 eV, calculated. For other phthalocyanine compounds; Co–Pc EHOMO = –5.1003 eV ELUMO = –2.9369 eV was calculated. Table 1 shows some chemical activity parameters. The molecule with a high dipole moment has a great asymmetry in the electronic charge distribution [38]. This situation is more reactive and sensitive and can change its electronic structure and its properties under a different interaction field. For compounds to have strong electron delocalization, they must have low electronegativity, high chemical potential, and low chemical hardness [39,40]. The chemical hardness of the compounds was in the range of 1.107–1.568 eV and the chemical softness was in the range of 0.540–0.784 eV. Therefore, it is understood that the compounds have good chemical stability [41].

**Table 1:** EHOMO, ELUMO, dipole moment (ρ, Debye), electronegativity (χ) and global electrophile (ω) etc. values of compounds (4-6).

Molecules Energy	Zn–Pc	Co–Pc	Cu–Pc
ELUMO	–2.6548	–2.9369	–3.1364
EHOMO	–4.8690	–5.1003	–5.3147
Energy gap (Δ)EHOMO- ELUMO	2.2142	2.1633	2.1782
Ionization potential (I = −EHOMO)	4.8690	5.1003	5.3147
Electron affinity (A = −ELUMO)	2.6548	2.9369	3.1364
Chemical hardness (η = (I − A)/2)	1.1071	1.0814	1.5682
Chemical softness (s = 1/2 η)	0.5535	0.5408	0.7841
Chemical potential (μ = (I + A)/2)	3.7619	4.0187	3.7465
Electronegativity (χ = (1+ A)/2)	1.8274	1.3576	2.0683
Electrophilicity index (ω= μ2/2 η)	6.6057	7.4650	4.4753
Dipole moment (μ)	4.8278	4.8699	9.2932

A dipole moment can be obtained from any standard electronic structure program. Hyperpolarizability, the nonlinear optical (NLO) property of a molecule, is quadratic electrical sensitivity per unit volume. Polarizations and hyperpolarizability characterize the return of a system in an applied electric field [38]. The dipole moment (independent of area, Debye) was calculated as Zn–Pc 4.8278, Co–Pc 4.8699, and Cu–Pc 9.2932. It is promised that these molecules will have nonlinear optical properties. The dipole moment values of zinc and cobalt phthalocyanine compounds were found close to each other. Copper phthalocyanine showed a higher dipole moment due to its electronic structure.

### 3.7. Molecular electrostatic potential (MEP)

MEP maps provide information about the electronic charge distribution of a molecule. The density of the electron distribution of the molecule is useful for illuminating bonds with descriptors such as polarity and electronegativity. The electronic structure and molecular reactivity of complex molecules can exhibit rich topographic properties [42].

**Figure 9:**
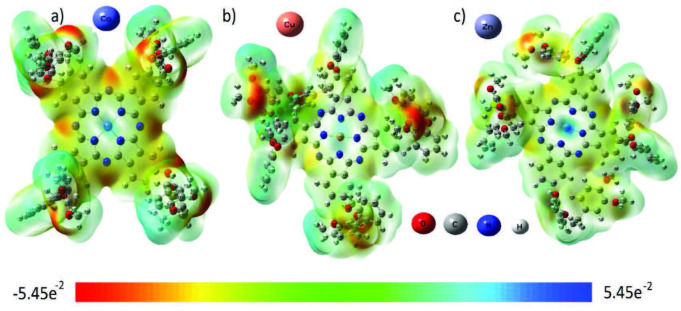
Molecular electrostatic potential mapped of compounds 4–6.

In this study, electrophilic potential (MEP) maps of three phthalocyanine molecules were obtained. As shown in Figure 9, it is visualized with MEP maps at the DFT/B3LYP/6-311G level using the GaussView 6.0.16 software. The MEP map shows that the region characterized by the blue color around the Zn, Co, and Cu atoms have positive values. The red regions on the map indicate the region rich in electrons. The aromatic ring region shows an almost neutral potential, most of which is represented by a yellow–green color [43]. Contour maps of phthalocyanines confirm negative and positive potential parameters in accordance with the electrostatic potential map (ESP). The phthalocyanine core in the structures shows a delocalized structure and high stabilization with green–yellow colors.

### 3.8. Photovoltaic properties

**Figure 10:**
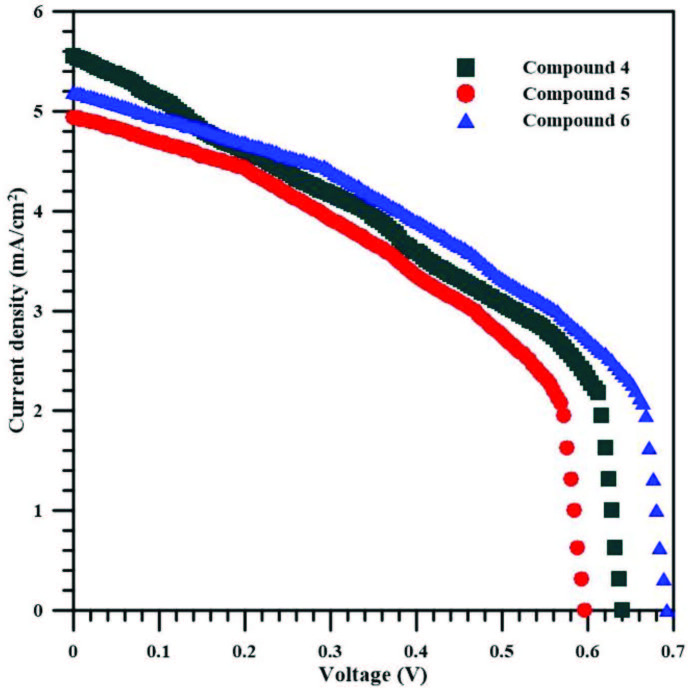
J-V curves of DSSCs based on phthalocyanine compounds 4–6.

The graph obtained as a result of the current voltage applied to the samples to examine the photovoltaic properties of the produced samples is given in Figure 10. JSC, VOC, Jmax, and Vmax values of the samples produced in DSSC structure, called compound 4, compound 5, and compound 6, were obtained from the current density (J) – voltage (V) graph, Figure 10. The fill factor (FF) and the energy conversion efficiency (η) of produced samples were calculated using the following Equation 1 and Equation 2, respectively [44].

(1)FF=ImaxVmaxISCVOC(2)η=ISCVOCPinFF

where P _in_ is the power of incident light. The values of FF and η are determined and indicated in Table 2 for all the produced samples. It can be seen that the HOMO and LUMO energy levels of the metal complexes used to form the DSSC structure are compatible with the valence band and the conduction band of TiO2. The observed Voc and Isc values of each sample can be indicated with the high number of electrons that flow into a conduction band of TiO2 from the excitement of compounds 4–6 by the absorption of the photon energy. These efficiencies of the samples show that the photovoltaic parameters of the DSSC structures are contributed by the metal complexes [45]. The results appear to be compatible with the studies in the literature [46]. Reasons such as limited reserves of fossil fuels and not being clean for the environment increase the demand for renewable energy sources. Here, the way to use solar energy economically is based on effective high power conversion efficient dye sensitive solar cells [47–50]. Dye sensitive solar cells made of phthalocyanine compounds, which are stable up to 400–500 degrees, provide the opportunity to benefit from solar energy, which is abundant, cheap, and clean. Among the reasons for the preference of phthalocyanines for this purpose, photochemical, thermal, and electrochemical stability are positive factors. Studies have reported that ruthenium polypyridyl complexes are the most common sensitizers in DSSCs, and the conversion efficiency here is about 12%. Moreover, ruthenium metal is limited for applications due to factors such as cost and environmental damage.

**Table 2:** The photovoltaic parameters for DSSCs based on phthalocyanine 4-6 complexes.

Samples	JSC (mA/cm2)	VOC (V)	FF	Η (%)
Compound 4	5.56	0.64	0.43	1.54
Compound 5	4.94	0.59	0.46	1.35
Compound 6	5.20	0.69	0.47	1.69

## 4. Conclusion

In this study, a new phthalonitrile derivative was synthesized. New zinc, cobalt, and copper phthalocyanine complexes were obtained from the reaction of synthesized starting material with metal salts. These compounds were characterized by general spectrophotometers. Then, fluorescence emission and absorption spectra and their properties were investigated. In addition to other properties of zinc phthalocyanine, fluorescence was characteristic. Furthermore, the compounds exhibited advantages such as nonaggregation behavior and good solubility in organic solvents. The structure of phthalocyanine molecules was examined using a 6–311 base set of DFT/B3LYP calculation method. Optimized bond lengths and angles were obtained, and it was determined that the molecular atom of the metal atom was placed planarly in the phthalocyanine nucleus. In addition, global reactivity parameters, HOMO and LUMO energy gaps that determine chemical stability, were calculated. Compounds 4–6 energy gap with soft molecular structure were calculated as close to each other as 2.2142 eV, 2.1633 eV, and 2.1782 eV, respectively. Dipole moments of Zn–Pc, Co–Pc, and Cu–Pc were calculated as 4.8278, 4.8699, and 9.2932 D. The results showed that the molecules have nonlinear optical properties. The solar conversion efficiency of DSSC devices based on copper, zinc, and cobalt phthalocyanine compounds was reported as 1.69%, 1.35%, and 1.54%, respectively. The measurement results showed that the compounds can be enriched with different additives for dye-sensitive solar cell technology.

Supplementary Materials
