# Supplementary material for: Design of novel substituted phthalocyanines; synthesis and fluorescence, DFT, photovoltaic properties
Source: Turk J Chem. 2020 Dec 16;44(6):1574–86. doi: 10.3906/kim-2007-40 (PMC7765769; doi:10.3906/kim-2007-40)
Supplement: Supplementary file 1 — Supplementary Materials [file turkjchem-44-1574-sup001.pdf]

Supplementary information

2. Experimental section

2.1. General

Hitachi U-2900 Spectrophotometer, Thermo Scientific FT-IR spectrophotometer, LC / MS (Thermo Fisher Scientific Inc., Waltham, MA, USA; TSQ-Quantum Access), Agilent 400 MHz spectrometer and Shimadzu RF-6000 spectrofluorophotometer devices were used for the structure characterization of the compounds (Agilent Technologies, Inc., Santa Clara, CA, USA; Shimadzu Corp., Kyoto, Japan). Chemicals and solvents were used commercially without purification.

2.2. 4-(2-(3,4-dimethoxyphenethoxy) phenoxy) phthalonitrile (3)

A mixture of 2-nitrophenol (0.402 g, 2.89 mmol) and 4-nitrophthalonitrile (0.500 g, 2.89 mmol) in 25 mL dimethylsulfoxide (DMSO) was stirred at room temperature under nitrogen atmosphere. After stirring for 15–20 min, 2-(3,4-dimethoxyphenyl) ethanol (0.527 g, 2.89 mmol) was added into the mixture. After stirring for 15 min, K_2CO_3 (2.2 g, 15.94 mmol) was added into the mixture over a period of 2 h. After this process, the stirring was stirred at 40 °C for a further 70 h. The reaction mixture was poured into ice water (150 mL) and precipitated.

It was filtered off, and washed with water to neutralize it. The product was dried in a vacuum oven at 80 °C. The product showed solubility in THF, DMSO, acetonitrile solvent. Yield; 0.47 g (40.61%). Mp: 132–135 °C. $C_{24}H_{20}$ N_2O_4: 400.43 g/mol. HRMS (ESI); (M+H) calc. for $C_{24}H_{20}$ N_2O_4: 400.43; found: 439.10 $[M+K]^+$. ^1H NMR (400 MHz, DMSO-d_6): (δ: ppm) 8.12, 8.01, 8.00, 7.74, 7.44, 6.90, 4.34, 3.72, 3.70, 3.31, 2.95, 2.48. ^{13}C NMR (400 MHz, DMSO-d_6): (δ: ppm) 162.27, 160.71, 149.11, 147.96, 146.67, 136.77, 136.49, 136.17, 130.40, 127.51, 126.81, 124.20,122.94,122.60,121.32,120.77,120.55, 116.72, 116.67, 116.16, 113.42, 112.36, 106.30, 70.13, 55.99, 55.92, 39.28. FT-IR spectrum (cm^{-1}): 3080(C–H aromatic),

2949, 2835, 2231(C≡N), 1600(C=C), 1517, 1467, 1442, 1301, 1249 (Ar–O–Ar), 1159, 1141, 1099, 1028, 948, 817, 752.

2.3. 2, 10, 16, 24 – Tetrakis 2-(3,4-dimethoxyphenethoxy) phenoxy phthalocyaninato) zinc (II) (4)

A mixture of 4-(2-(3,4-dimethoxyphenethoxy) phenoxy) phthalonitrile **3** (0.050 g**,** 0.0297 mmol) and $Zn(CH_3COO)_2$ (0.023 g) was powdered in a quartz crucible and heated for 5 min 230 ºC in a sealed glass tube. After reaching room temperature, the product was washed with hot and cold water, ethanol, and methanol. The product soluble in THF was collected and the solvent was removed to obtain a green solid. This compound showed solubility in dichloromethane, $CHCl_3$, THF, DMF, DMSO solvents. Yield: 56.00%. HRMS (ESI); (M+H) calc. for $C_{97}H_{83}N_8O_{16}Zn$: 1682.12; found: 1705.50 $[M+Na]^+$. UV-Vis (THF) λmax (log ε): 676 (5.32), 610(4.81), 348 (5.17). ^1H NMR (400 MHz, DMSO-d6) δ ppm: 9.22, 8.81, 7.74, 7.15, 7.03, 6.99, 6.98, 6.85, 4.73, 3.76, 3.70, 3.41, 3.36, 3.31, 3.25, 2.48, 1.34. IR spectrum (cm^{-1}): 3100, 2964, 2835, 1598, 1477, 1261, 1232, 1139, 1089, 1026, 952, 808.

2.4. 2, 10, 16, 24 – Tetrakis 2-(3,4-dimethoxyphenethoxy) phenoxy phthalocyaninato) cobalt (II) (5)

This compound was synthesized under the same conditions of phthalocyanine compound **4**, except for the metal salt ($CoCl_2$) used. Yield: 48.00%. HRMS (ESI); (M+H) calc. for $C_{97}H_{83}N_8O_{16}Co$: 1675.67; found: 1698.76 $[M+Na]^+$. UV-Vis (THF) λmax (log ε): 664 (5.25), 326 (5.20). IR spectrum (cm^{-1}): 3078, 2927, 2831, 1606, 1514, 1462, 1409, 1261, 1232, 1192, 1124, 1093, 1058, 1012, 954, 848.

2.5. 2, 10, 16, 24 – Tetrakis 2-(3,4-dimethoxyphenethoxy) phenoxy phthalocyaninato) copper (II) (6)

This compound was synthesized under the same conditions of phthalocyanine compound **4**, except for the metal salt ($CuCl_2$) used. Yield: 48.00%. HRMS (ESI); (M+H) calc. for $C_{97}H_{83}N_8O_{16}Cu$: 1680.29; found: 1703.40 $[M+Na]^+$. UV-Vis (THF) λmax (log ε): 676 (5.23), 610(4.77), 340 (5.09). IR spectrum (cm^{-1}): 3080, 2966, 2879, 1600, 1514, 1463, 1259, 1234, 1138, 1026, 927,806.

2.6. Theoretical analysis

All calculations were made using DFT calculations according to the basis set of B3LYP / 6-311G. Conformational analysis was performed with semiempirical method on PM3 set. In the next step, the values obtained with basis set 6-311 of DFT / B3LYP method were used to calculate minimum energy and bond lengths. Molecular structure, energies, NBO analysis, MESP maps of optimized geometries of M-Pc were calculated using Gaussian 09 and GaussView 6.0 package program [1-2].

2.7. The current density (J) - voltage (V) measurement

In conventional dye sensitive solar cell (DSSC) structures, fluorine doped tin oxide (FTO) coated glass substrates were used as working electrodes [3–7]. TiO_2 nanopowder, which was put into paste with polyethylene glycol (PEG300) solution, was coated on the standard cleaned substrates with the doctor blade technique. Fields covered with TiO_2 have a surface area of ~0.2 cm^2 and a thickness of ~50 μm. After coating process, the samples were annealed in a quartz furnace at 450 °C for 15 min. By using dimethylformamide (DMF) as solvent, 1 mM of phthalocyanine (Pc) solutions were dropped to the samples to obtain dye sensitive of TiO_2. For the samples expected to dry for 24 h at room temperature, the dripping process was repeated 3 times to ensure a tight covalent bond between TiO_2 and phthalocyanines. Commercially available an I^-/I_3^- electrolyte containing 50 mM iodide/tri-iodide was dropped on three samples ready to make contact and combined with platinum coated FTO glasses. The current–voltage

(I–V) measurement of samples was achieved under solar simulator with an AM1.5 filter and 100 mW/cm^2 illumination.

References

1. Frisch GWTMJ, Schlegel HB, Scuseria GE, Robb MA, Cheeseman JR et al. Gaussian 09, Revision A.02. Wallingford, CT, USA: Gaussian, Inc., 2016.
2. Chindeka F, Mashazi P, Britton J, Fomo G, Oluwole DO et al. Optimizing phthalocyanine based dye-sensitized solar cells: the role of reduced graphene oxide. Synthetic Metals 2018; 246: 236-245.
3. O'Regan B, Grätzel M. A low-cost, high-efficiency solar cell based on dye-sensitized colloidal TiO_2 films. Nature 1991; 353: 737-740.
4. Komori T, Amao Y. Dye-sensitized solar cell with the near-infrared sensitization of aluminum phthlocyanine. Journal of Porphyrins and Phthlocyanines 2003; 7: 131-136.
5. Yan X, Fan H, Gu H, Zhang J, Huang et al. Synthesis of an octathienyl-fused phthalocyanine as a donor material for organic solar cells. Dyes and Pigments 2015; 114: 124-128.
6. Polat MP, Yenilmez HY, Koca A, Altındal A, Bayır ZA. Metallophthalocyanines bearing four 3-(pyrrol-1-yl) phenoxy units as photosensitizer for dye-sensitized solar cells. Dyes and Pigments 2018; 156: 267-275.
7. Sevim AM, Çakar S, Özacar M, Gül A. Electrochemical and photovoltaic properties of highly efficient solar cells with cobalt/zinc phthalocyanine sensitizers. Solar Energy 2018; 160: 18-24.

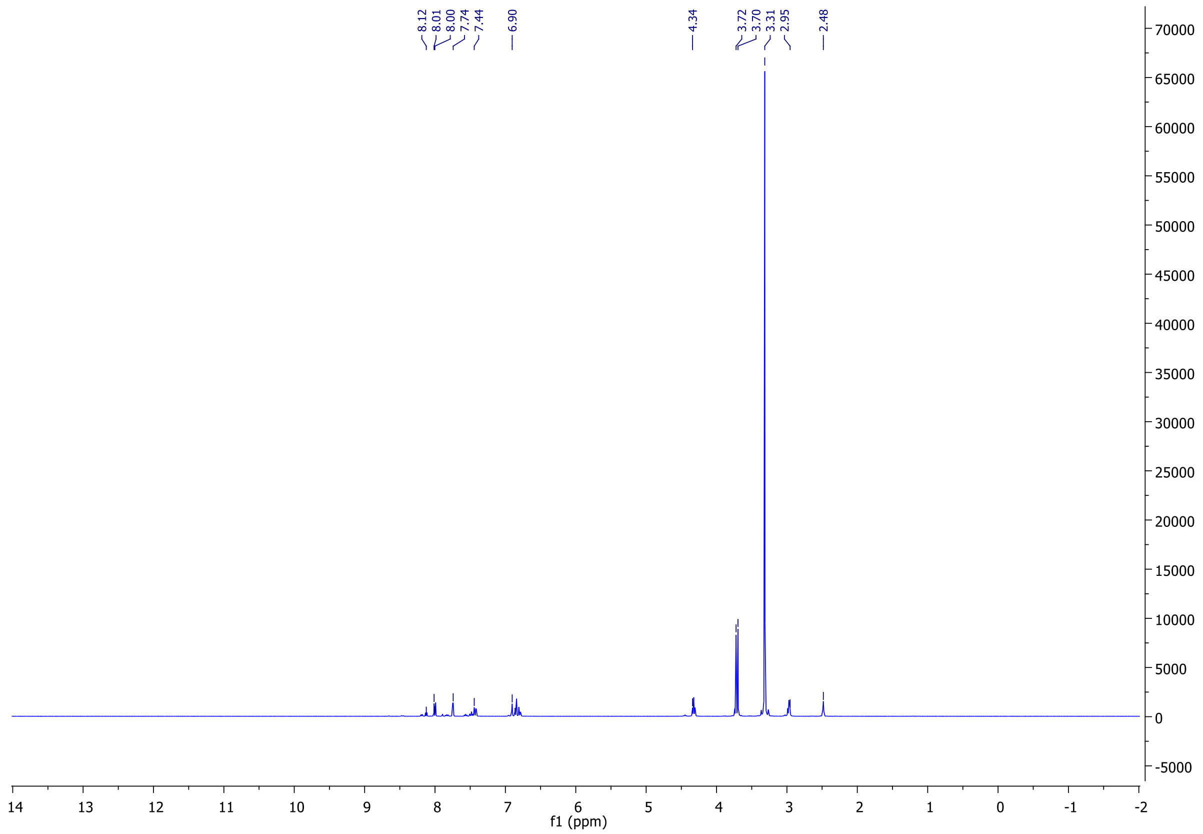

Figure S1. ^{1}H-NMR spectra of compound **3**.

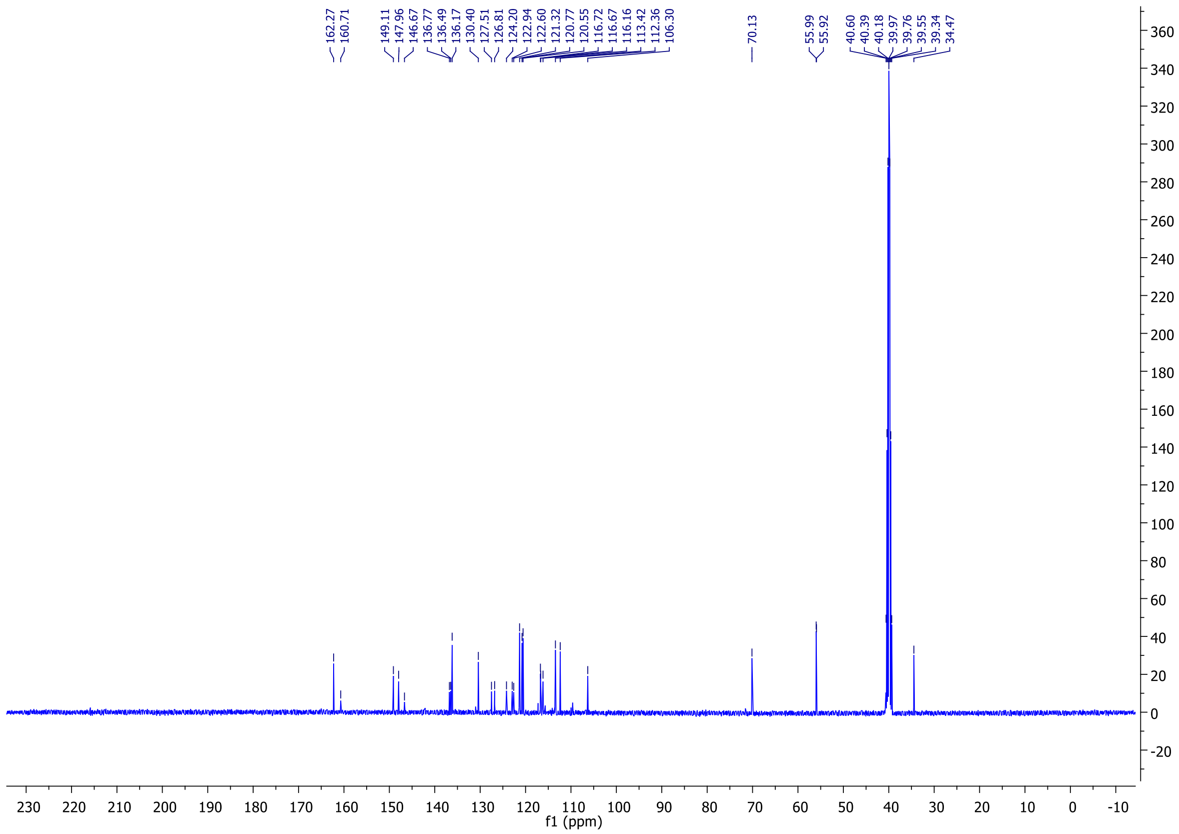

Figure S2. ^{13}C-NMR spectra of compound **3**.

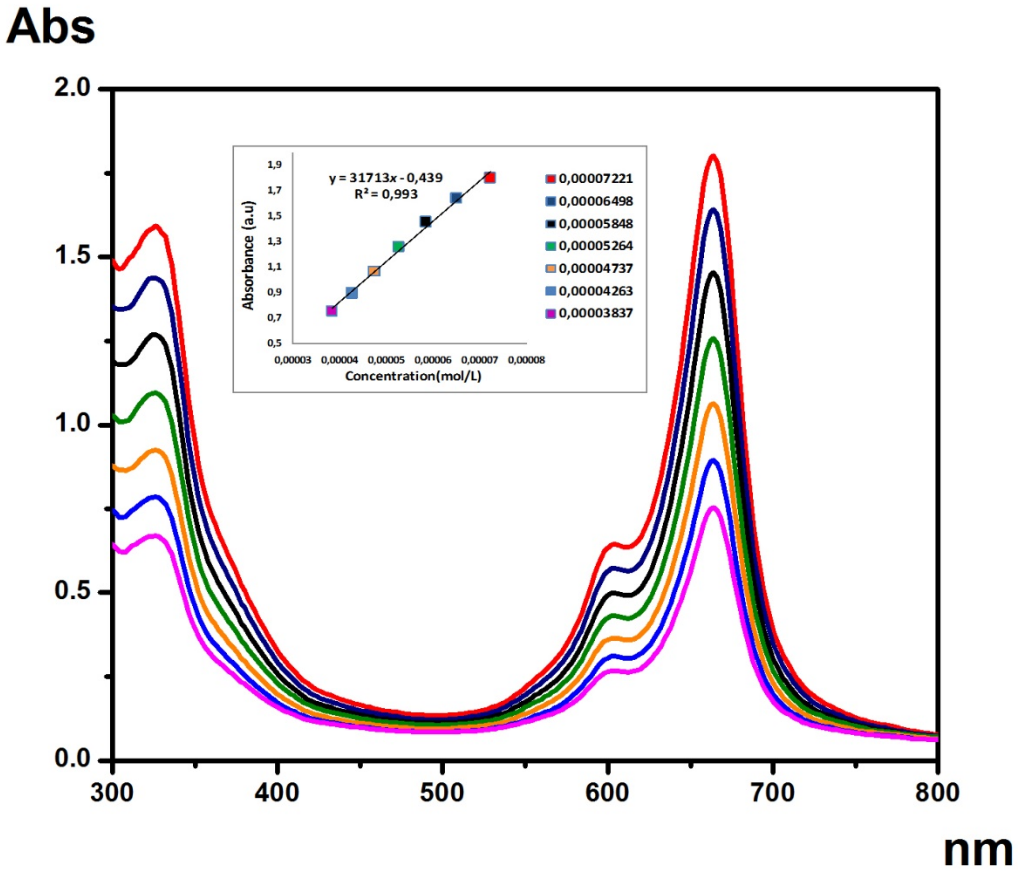

Figure S3. Electronic absorption of compound **5** in different concentrations.

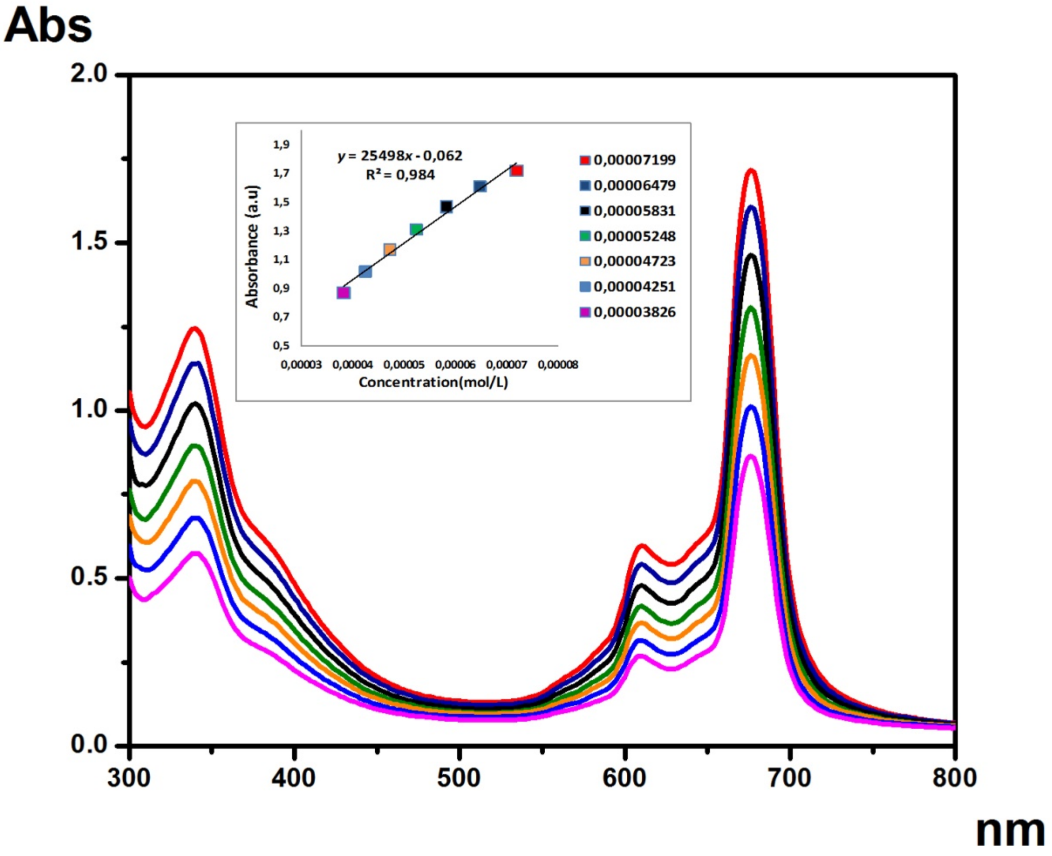

Figure S4. Electronic absorption of compound **6** in different concentrations.

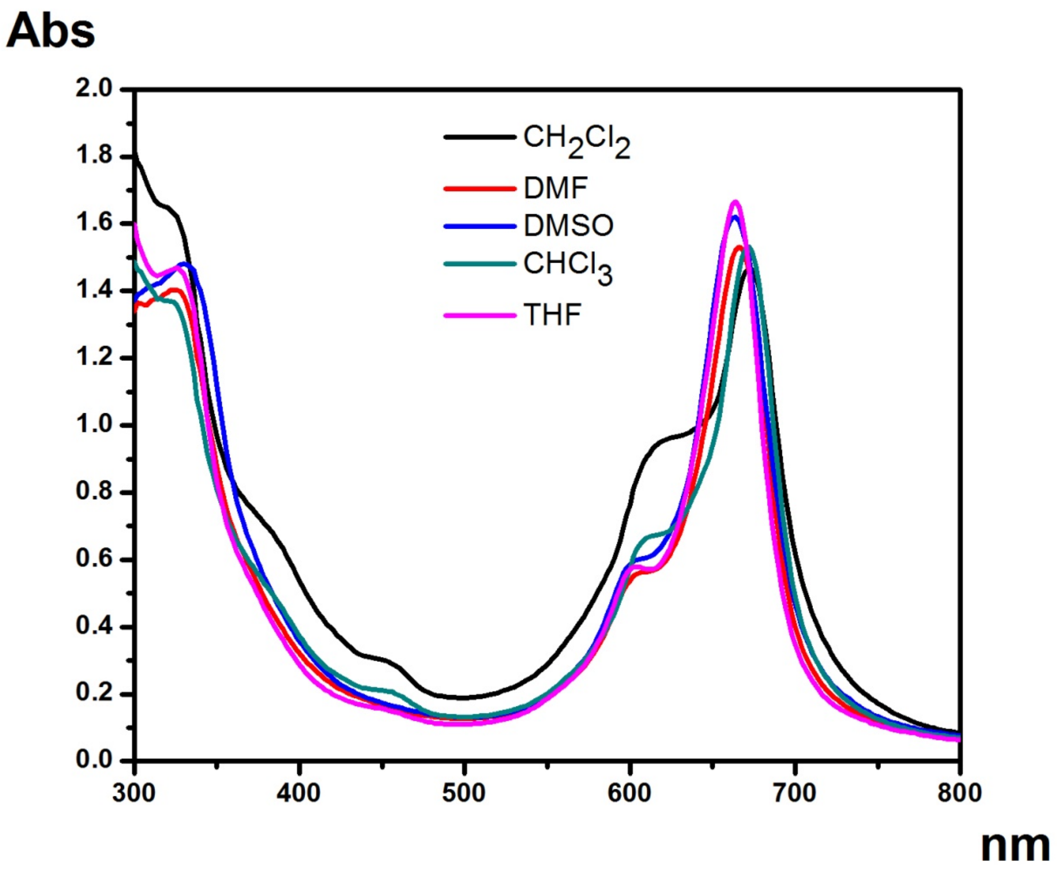

Figure S5. Electronic absorption of compound **5** in different solvents.

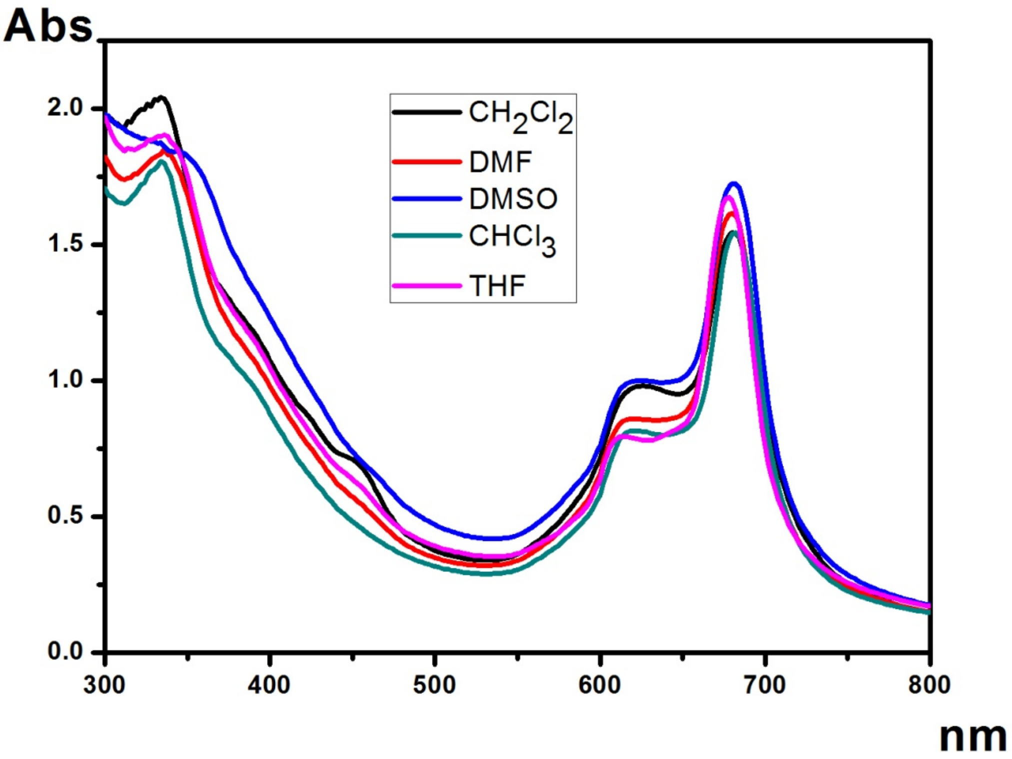

Figure S6. Electronic absorption of compound **6** in different solvents.
